# Learning to Monitor Machine Health with Convolutional Bi-Directional LSTM Networks

**DOI:** 10.3390/s17020273

**Published:** 2017-01-30

**Authors:** Rui Zhao, Ruqiang Yan, Jinjiang Wang, Kezhi Mao

**Affiliations:** 1 School of Instrument Science and Engineering, Southeast University, Nanjing 210009, China; RZHAO001@e.ntu.edu.sg; 2 School of Electrical and Electronic Engineering, Nanyang Technological University, Singapore 639798, Singapore; ekzmao@ntu.edu.sg; 3 School of Mechanical and Transportation Engineering, China University of Petroleum, Beijing 102249, China; jwang@cup.edu.cn

**Keywords:** machine health monitoring, tool wear prediction, convolutional neural network, recurrent neural network, bi-directional long-short term memory network

## Abstract

In modern manufacturing systems and industries, more and more research efforts have been made in developing effective machine health monitoring systems. Among various machine health monitoring approaches, data-driven methods are gaining in popularity due to the development of advanced sensing and data analytic techniques. However, considering the noise, varying length and irregular sampling behind sensory data, this kind of sequential data cannot be fed into classification and regression models directly. Therefore, previous work focuses on feature extraction/fusion methods requiring expensive human labor and high quality expert knowledge. With the development of deep learning methods in the last few years, which redefine representation learning from raw data, a deep neural network structure named Convolutional Bi-directional Long Short-Term Memory networks (CBLSTM) has been designed here to address raw sensory data. CBLSTM firstly uses CNN to extract local features that are robust and informative from the sequential input. Then, bi-directional LSTM is introduced to encode temporal information. Long Short-Term Memory networks (LSTMs) are able to capture long-term dependencies and model sequential data, and the bi-directional structure enables the capture of past and future contexts. Stacked, fully-connected layers and the linear regression layer are built on top of bi-directional LSTMs to predict the target value. Here, a real-life tool wear test is introduced, and our proposed CBLSTM is able to predict the actual tool wear based on raw sensory data. The experimental results have shown that our model is able to outperform several state-of-the-art baseline methods.

## 1. Introduction

During recent years, machine monitoring systems, including diagnosis and prognosis approaches, have been actively researched [1,2,3,4]. Diagnosis and prognosis systems focus on the detection of faults after the occurrence of certain faults and predictions of the future working conditions and the Remaining Useful Life (RUL), respectively [5,6,7,8,9,10,11]. The methodologies behind these existing machine monitoring systems can be divided into two major categories: physics-based and data-driven models [12,13]. In physics-based models, domain knowledge of physical models and laws with measured data are incorporated into a model constructed via mathematical equations. Previous proposed models, including the Paris crack growth model [14], the Forman crack growth model [15], and so on, all belong to physics-based ones. However, the physics-based models have been criticized for these following points. Firstly, the performance of physics-based models is heavily dependent on the quality and accuracy of domain knowledge about the practical mechanical systems. In real life, due to complexity and noisy working conditions, such a kind of high-quality domain knowledge is often unavailable, which hinders the robustness of these physics-based models. Secondly, most of physics-based models are unable to be updated with on-line measured data, which limits the effectiveness and flexibility of the applications of physics-based models. On the other hand, data-driven models focus on modeling based on historical measured data and try to make a decision from the online data collected from sensors on working machines. Additionally, these model parameters can be updated in real time when the working status of the machines changes. In addition, the development of advanced sensors and computing systems make the research topic of data-driven machine monitoring systems more and more attractive. Therefore, in this paper, our work focuses on this data-driven framework.

**Figure 1:**
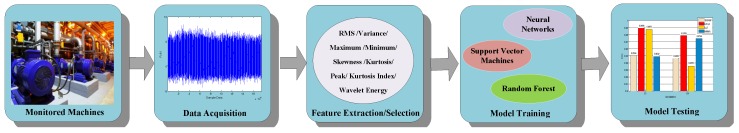
Framework of data-driven machine monitoring systems.

As shown in Figure 1, the basic pipeline framework behind data-driven models consists of four major parts: data acquisition, feature extraction/selection, model training and model testing. These systems take various sensor data as inputs and perform feature selections and extractions to derive representation of machine conditions. Then, representations are fed into various algorithms, which normally consist of two parts: one is model training based on historical data, and the other is model prediction based on current sampled data. The core step in data-driven models is representation learning of these sensor data. Sensor data are in nature time series data, which are sampled by sensors and expressed in a sequential form. Some previous works focus on multi-domain feature extractions, including statistical (variance, skewness, kurtosis), frequency (spectral skewness) and time frequency (wavelet coefficients) features. However, these methods do not belong to sequence models, which cannot model the intrinsic sequential characteristic behind sensory data. Additionally, how to select these features is another big challenge for these methods. These models require intensive expert knowledge or feature engineering. Except these methods based on hand-engineered features, some sequence models, including Markov models, Kalman filters and conditional random fields, are powerful for addressing sequential data, which only access raw time series [16,17,18]. However, they have been criticized for the inability to capture long-range dependencies. In sensory data for machine monitoring, two informative and discriminative signals may be separated by many indiscriminative or even noisy signals representing a long time period. Therefore, the long delays that separate some important samples in the time scale may lead to failures of these above sequences models. During recent years, Recurrent Neural Networks (RNNs), especially Long Short-Term Memory (LSTM), that were proposed to relieve the problem of gradient exploding or vanishing in RNN, have emerged as one popular architecture to handle sequential data with various applications, including image captioning, speech recognition, genomic analysis and natural language processing [19,20,21]. LSTM is able to address sequences of varying length data and capture long-term dependencies. As one kind of neural network, LSTM incorporates representation learning and model training together, which require no additional domain knowledge. Additionally, this structure can enable us to discover some unseen structure to improve the generalization capability of the model. Except the necessity of temporal information, raw sensory data usually contain noise. The LSTM models built on top of raw sensory data may not be robust. Therefore, Convolutional Neural Networks (CNN) are introduced to extract local features. The core idea of CNN lies in that abstract features can be extracted by convolutional kernels and the pooling operation. In CNN, the convolutional layers (convolutional kernels) convolve multiple local filters with raw sequential data and generate invariant local features, and the subsequent pooling layers extract the most significant features within fixed length sliding windows. Here, we adopt CNN to extract a sequence of local features from the raw signal firstly.

In this paper, we combine CNN with bi-directional LSTM to propose a novel machine health monitoring system named Convolutional Bi-directional LSTM networks (CBLSTMs). In our proposed CBLSTMs, CNN can extract local robust features, and bi-directional LSTMs, which are built on CNN, are able to encode the temporal information and learn representations. Different from conventional LSTMs that process the input sequence in a feedforward manner, bi-directional LSTMs model the input sequence forward and backward [22]. The core idea behind bi-directional LSTM is that each sequence is presented forwards and backwards to two separate LSTMs, and bi-directional LSTMs can access complete, sequential information about all context information before and after each time step in a given sequence. Here, we adopt one open source dataset: dynamometer, accelerometer and acoustic emission data sampled from high-speed Computer Numerical Control (CNC) milling machine cutters (the dataset has been kindly provided at https://www.phmsociety.org/competition/phm/10). The corresponding task is defined as the estimation of tool wear conditions based on sensory signals, i.e., tool wear depth [23,24]. In our setting, this problem has been transformed into a regression problem with sequential data, in which each sequential datum, i.e., sensory data, represents one certain tool wear condition that corresponds to the actual tool wear width. Several state-of-the-art models are compared with our proposed CBLSTMs model.

This paper is organized as follows. In Section 2, some related work including CNN, LSTM and their various applications, are reviewed. Then, our proposed CBLSTMs are presented in Section 3. Then, in the following Section 4, experimental results of the prediction of tool wear condition are illustrated. Finally, concluding remarks are provided in Section 5.

## 2. Related Work

### 2.1. Convolutional Neural Network

CNNs were firstly proposed by LeCun [25] for image processing, which is featured by two key properties: spatially-shared weights and spatial pooling. CNNs have shown their success in various computer vision applications [25,26,27,28] for which the input data are usually 2D data. CNN has also been introduced to address sequential data, including natural language processing and speech recognition [29,30,31]. Generally, to address sequences, the 1D convolutional layer in CNN firstly adopts multiple local filters over the whole sequential input. Each feature map corresponding to each local filter can be generated by sliding the filter over the sequence. Then, the following pooling layer is applied to extract the most vital and fixed-length features from each feature map. Additionally, both convolution and pooling layers can be performed in a stacked way.

In our proposed CBLSTM network, CNN is firstly adopted to process time series data. Then, the outputs of CNN model are then fed into the following bi-directional LSTMs. It is believed that CNN is able to encode more critical information compared to the raw sequential input, considering that the single time step information may not be discriminative enough. In addition, CNN is able to compress the length of the sequence, which increases the capability of the subsequent recurrent models to capture temporal information.

### 2.2. From RNN to LSTM

Recurrent Neural Networks (RNNs) were proposed for sequence learning. RNNs build connections between units from a directed cycle. Different from the basic neural network, multi-layer perceptron, which can only map from input data to target vectors, RNN is able to map target vectors from the entire history of previous inputs in principal. RNN allows a memory of previous inputs to be kept in the network’s internal state. RNNs can be trained via backpropagation through time for supervised tasks. However, the vanishing gradient problem during backpropagation for model training hinders the performance of RNN. This means that traditional RNN may not capture long-term dependencies. Therefore, LSTMs were firstly presented to prevent backpropagated errors from vanishing or exploding. Forget gates were introduced in LSTMs to avoid the long-term dependency problem. These adopted forget gates are able to control the utilization of information in the cell states. To capture nonlinear dynamics in time series sensory data and learn effective representation of machine conditions, LSTMs should be superior compared to traditional RNNs due to their capability to capture long-term dependencies. Considering that LSTMs are able to capture long-range dependencies and nonlinear dynamics in time series data, LSTMs have been successfully applied in various applications, including speech recognition, image captioning, handwriting recognition, genomic analysis and natural language processing.

Our proposed CBLSTM utilizes bi-directional LSTM to model temporal information. Bi-directional LSTM processes input sequences in forward and backward directions and is able to summarize temporal information from past and future contexts. Adopting the bi-directional structure, the past and future dependency information are both exploited to capture the temporal information.

### 2.3. Neural Network for Machine Health Monitoring

Due to the strong representation capability of multi-layer neural networks, neural networks have been widely applied to machine health monitoring problems [32,33,34,35,36,37,38,39,40]. Most of the previous works do not consider the sequential nature behind sensory data. Before feeding raw data into the neural network, feature extraction and selection are performed firstly [32,33,34,35,41,42]. These works do not consider the order of the signal and require some feature engineering. After wavelet techniques, CNN has been applied on the time frequency map of time series data for fault diagnosis [38]. Compared to the previous CNN model, CNN in our proposed CBLSTM only takes raw sensory data as input. Additionally, CBLSTM utilizes CNN to extract local features instead of the final representation of the whole sequence. In addition, some papers have applied RNNs including LSTMs to machine health monitoring problems [36,37,39]. In this paper, we further proposed bi-directional LSTMs combined with CNN to address machine health monitoring problems.

## 3. Models

Before the presentation of our proposed CBLSTMs, some adopted notations in this paper are introduced firstly. In this paper, machine health monitoring problems are cast into a specific one: the tool wear prediction problem. The in-process sensory data as time series observations are used as input data. The task is defined as designing a model to infer the tool wear conditions from these in-process multi-sensory signals. Let a series of observations $x_{i}=\left[x_{i}^{\left(1\right)},\cdots ,x_{i}^{\left(l\right)}\right]$ denote the acquired data for the *i*-th machine condition sample. Additionally, $x_{i}^{\left(t\right)}\in R^{d}$ represents the multi-sensory data sampled at time step *t*, which is a vector, and *d* is the dimensionality of sensory data. *l* is the length of the sensory signal. For each sequential datum $x_{i}$, the corresponding actual tool wear condition (flank wear width) is measured and recorded as $y_{i}$. The tasks are defined to predict ${\bar{y}}_{i}$ based on sequential sensory data $x_{i}$.

**Figure 2:**
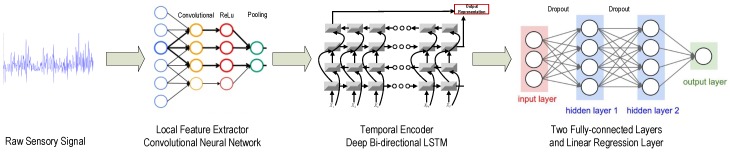
Framework of our proposed Convolutional Bi-directional Long Short-Term Memory networks (CBLSTM).

Our proposed CBLSTMs consists of two major parts: one is the local feature extractor, CNN, and the other one is the temporal encoder, bi-directional LSTMs. After applying one-layer CNN on the raw input sequence to extract local and discriminative features, two-layer bi-directional LSTMs are built on top of the previous CNN to encode the temporal patterns. Then, two fully-connected dense layers are stacked together to process the outputs of LSTMs. Finally, a linear regression layer is adopted to predict the tool wear depth. The whole structure of the proposed CBLSTM is shown in Figure 2.

### 3.1. Local Feature Extractor: CNN

The adopted one-layer CNN consists of two layers: one convolutional layer and one pooling layer, which directly process the raw input sequence. The convolutional layer slides the filters over the whole input sequence to generate feature maps. Each feature map can be regarded as the convolutional activation of the corresponding filter over the whole sequence. It is assumed that *k* filters with a window size of *m* are used in the convolutional layer. Then, the pooling layer is applied to compress each generated feature map to produce significant features. The details of these two layers are presented in the following subsections:

Convolution: The convolution operation is defined as a multiplication operation between a filter vector $u\in R^{md}$ and a concatenation vector representation $x_{i:i+m-1}$ given by:

(1) $$x_{i:i+m-1}=x_{i}⊕x_{i+1}⊕\cdots ⊕x_{i+m-1}$$

where $x_{i:i+m-1}$ represents a window of *m* continuous time steps starting from the *i*-th time step. In addition, a bias term *b* is also added into the convolution operation, so that the final operation is given as:

(2) $$c_{i}=g\left(u^{T}x_{i:i+m-1}+b\right)$$

where ${*}^{T}$ denotes the transpose of a matrix * and *g* is a non-linear activation function that is set to Rectified Linear Units(ReLu)in our model [43].

Each vector u can be regarded as a filter, and the single value $c_{i}$ can be regarded as the activation of the window.

Max-pooling: The convolution operation over the whole sequence is applied by sliding the filtering window from the beginning time step to the ending time step. It is easily shown that a feature map is a vector denoted as follows:

(3) $$c_{j}=\left[c_{1},c_{2},\cdots ,c_{l-m+1}\right]$$

where the index *j* denotes the *j*-th filter. It corresponds to multi-windows as $\left\{x_{1:m},x_{2:m+1},\cdots ,x_{l-m+1:l}\right\}$. The pooling layer is able to reduce the length of the feature map, which can further minimize the number of model parameters. The hyper-parameter of pooling layer is the pooling length denoted as *s*. The max operation is taking a max over the *s* consecutive values in feature map $c_{j}$.

Then, the compressed feature vector can be obtained as:

(4) $$h=\left[h_{1},h_{2},\cdots ,h_{\frac{l-m}{s}+1}\right]$$

where $h_{j}=\max\left(c_{(j-1)s},c_{(j-1)s+1},\cdots ,c_{js-1}\right)$. Generally, multiple filters are applied with different initialized weights to derive the output of the CNN layer.

**Figure 3:**
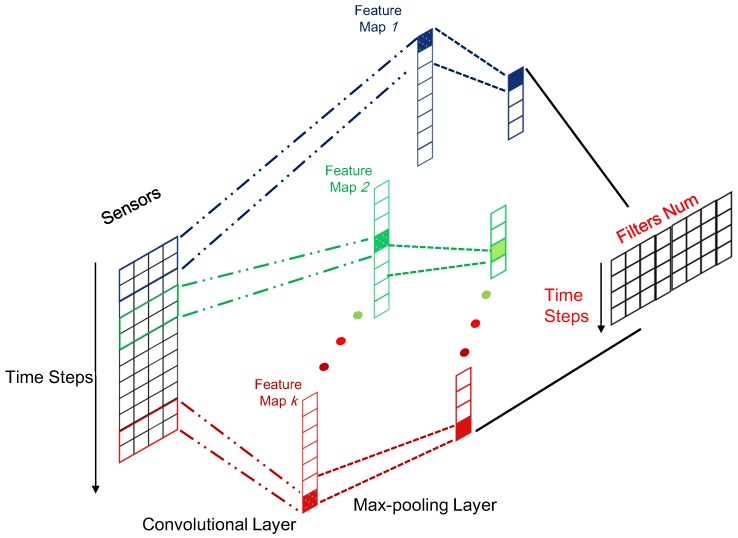
Illustrations for the local feature extractor.

Generally, the size of the input sequence in the CNN layer is n×l×d, and *n* is the number of data samples. The size of the corresponding outputs is $n\times (\frac{l-m}{s}+1)\times k$. It is easily shown that after the convolutional and pooling operation, the length of sequence data can be compressed from *l* to $\left(\frac{l-m}{s}+1\right)$. Compared to the original representation is raw sensory data with a dimensionality of *d* that is usually the number of sensors in each time step; more abstract and informative representation can be learned after CNN, and the corresponding dimensionality is *k*, which is the number of filters. Therefore, CNN plays the role of feature extractor to feed better sequential representation into the subsequent LSTM models compared to the raw sequential data. To give a clear illustration, the framework for the local feature extractor based on CNN has been displayed in Figure 3.

### 3.2. Temporal Encoder: Bi-Directional LSTMs

Here, a two-layer bi-directional LSTM is built on the top of the CNN. This recurrent model is applied to summarize the temporal information. After introducing the basic LSTMs, deep bi-directional LSTM is presented.

#### 3.2.1. Basic LSTMs

The core idea behind LSTMs lies in that at each time step, a few gates are used to control the passing of information along the sequences that can capture long-range dependencies more accurately. In our paper, we adopt one popular LSTM framework proposed in [44]. In LSTM, at each time step *t*, hidden state $h^{t}$ is updated by current data at the same time step $x^{t}$, the hidden state at the previous time step $h^{t-1}$, the input gate $i^{t}$, the forget gate $f^{t}$, the output gate $o^{t}$ and a memory cell $c^{t}$. The following updating equations are given as follows:

(5) $$\begin{array}{c} i^{t}=\sigma (W^{i}x^{t}+V^{i}h^{t-1}+b^{i}),\\ f^{t}=\sigma (W^{f}x^{t}+V^{f}h^{t-1}+b^{f}),\\ o^{t}=\sigma (W^{o}x^{t}+V^{o}h^{t-1}+b^{o}),\\ c^{t}=f^{t}⊙c^{t-1}+i^{t}⊙\tanh\left(W^{c}x^{t}+V^{c}h^{t-1}+b^{c}\right),\\ h^{t}=o^{t}⊙\tanh\left(c^{t}\right). \end{array}$$

where model parameters including all $W\in R^{d\times k}$, $V\in R^{d\times d}$ and $b\in R^{d}$ are shared by all time steps and learned during model training, *σ* is the sigmoid activation function, ⊙ denotes the element-wise product and *k* is a hyper-parameter that represents the dimensionality of hidden vectors. Here, Equation (5) defines the hidden layer function H.

**Figure 4:**
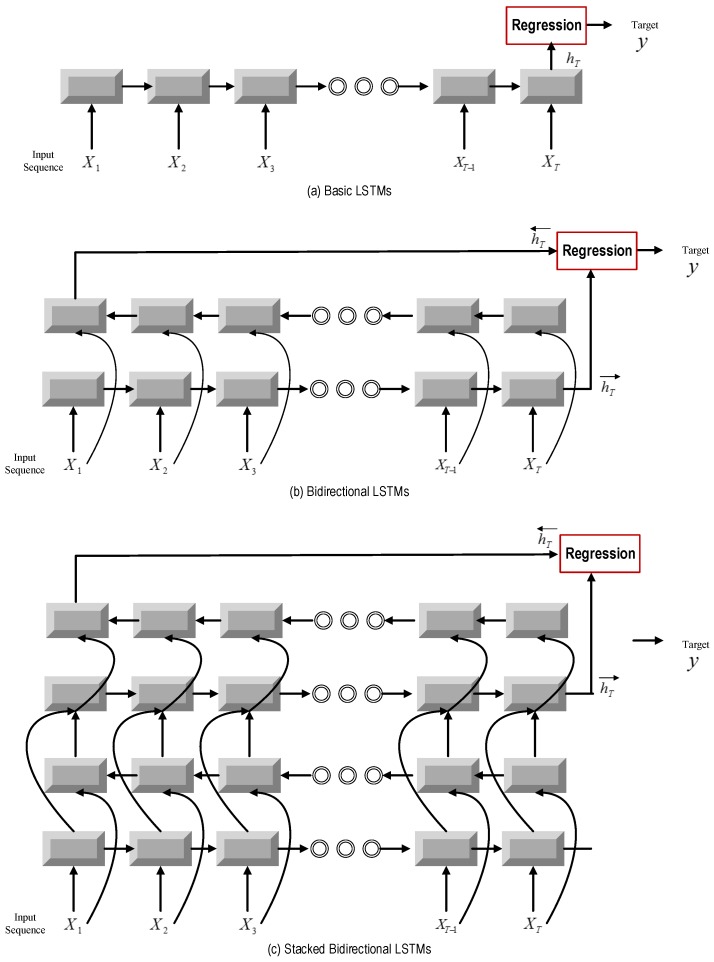
Illustrations for basic LSTMs and the three-layer stacked LSTM model for the sequential data regression problem. The grey blocks denote an LSTM’s layer, while dark red blocks represent a linear regression layer.

Firstly, the basic LSTM is constructed to process the sequential data in time order. Additionally, the output at the terminal time step is used to predict the output by a linear regression layer, as shown in the following equation.

(6) $${\bar{y}}_{i}=W^{r}h_{i}^{T}$$

where $W^{r}\in R^{k\times z}$ and *z* is the dimensionality of the output. In our tasks, the output is the flank wear width, so that z=1. For model training, the predicted tool wear value $\bar{y}$ is compared with the true tool wear value *y* to derive the Mean Squared Error (MSE) as model loss.

(7) $$\mathrm{loss}=\frac{1}{n}\sum_{i=1}^{n}{\left(\bar{y_{i}}-y_{i}\right)}^{2}$$

where *n* is the training sample size. The corresponding LSTMs architecture is shown in Figure 4a.

#### 3.2.2. Bi-Directional LSTMs

It is easily shown that basic LSTMs are only able to access the previous context of each specific time step. However, in machine health monitoring systems, the sequential sensory data have strong temporal dependencies. It is meaningful to consider the future context. Therefore, the bi-directional LSTM is applied here. Bi-directional LSTMs are able to process the sequence data in two directions including forward and backward ways with two separate hidden layers and then feed forward to the same output layer. The following equations define the corresponding hidden layer function, and the → and ← denote the forward and backward process, respectively.

(8) $$\begin{array}{c} {\vec{i}}^{t}=\sigma ({\vec{W}}^{i}{\vec{x}}^{t}+{\vec{V}}^{i}{\vec{h}}^{t-1}+{\vec{b}}^{i}),\\ {\vec{f}}^{t}=\sigma ({\vec{W}}^{f}{\vec{x}}^{t}+{\vec{V}}^{f}{\vec{h}}^{t-1}+{\vec{b}}^{f}),\\ {\vec{o}}^{t}=\sigma ({\vec{W}}^{o}{\vec{x}}^{t}+{\vec{V}}^{o}{\vec{h}}^{t-1}+{\vec{b}}^{o}),\\ {\vec{c}}^{t}={\vec{f}}^{t}⊙{\vec{c}}^{t-1}+{\vec{i}}^{t}⊙\tanh\left({\vec{W}}^{c}{\vec{x}}^{t}+{\vec{V}}^{c}{\vec{h}}^{t-1}+{\vec{b}}^{c}\right),\\ {\vec{h}}^{t}={\vec{o}}^{t}⊙\tanh\left({\vec{c}}^{t}\right). \end{array}$$

(9) $$\begin{array}{c} {\overset{\leftarrow }{i}}^{t}=\sigma ({\overset{\leftarrow }{W}}^{i}{\overset{\leftarrow }{x}}^{t}+{\overset{\leftarrow }{V}}^{i}{\overset{\leftarrow }{h}}^{t+1}+{\overset{\leftarrow }{b}}^{i}),\\ {\overset{\leftarrow }{f}}^{t}=\sigma ({\overset{\leftarrow }{W}}^{f}{\overset{\leftarrow }{x}}^{t}+{\overset{\leftarrow }{V}}^{f}{\overset{\leftarrow }{h}}^{t+1}+{\overset{\leftarrow }{b}}^{f}),\\ {\overset{\leftarrow }{o}}^{t}=\sigma ({\overset{\leftarrow }{W}}^{o}{\overset{\leftarrow }{x}}^{t}+{\overset{\leftarrow }{V}}^{o}{\overset{\leftarrow }{h}}^{t+1}+{\overset{\leftarrow }{b}}^{o}),\\ {\overset{\leftarrow }{c}}^{t}={\overset{\leftarrow }{f}}^{t}⊙{\overset{\leftarrow }{c}}^{t+1}+{\overset{\leftarrow }{i}}^{t}⊙\tanh\left({\overset{\leftarrow }{W}}^{c}{\overset{\leftarrow }{x}}^{t}+{\overset{\leftarrow }{V}}^{c}{\overset{\leftarrow }{h}}^{t+1}+{\overset{\leftarrow }{b}}^{c}\right),\\ {\overset{\leftarrow }{h}}^{t}={\overset{\leftarrow }{o}}^{t}⊙\tanh\left({\overset{\leftarrow }{c}}^{t}\right). \end{array}$$

Then, the complete BLSTM hidden element representation $h^{t}$ is the concatenated vector of the outputs of forward and backward processes as follows:

(10) $$h^{t}={\vec{h}}^{t}⊕{\overset{\leftarrow }{h}}^{t}$$

Deep bi-directional LSTM: During recent years, deep architectures have been shown to be successful in representation learning [45,46]. Therefore, it is meaningful to stack multiple LSTM layers to form a deep LSTM neural network. The core idea behind the deep neural network is that inputs to the model should go through multiple non-linear layers. When it comes to deep LSTMs, the input to the model can be passed through multiple LSTM layers. As shown in Figure 4c, the hidden output of one LSTM layer is not only propagated through time, but also used as the input data to the next LSTM layer. The output sequence of one layer is fed into the next layer. In the framework of bi-directional LSTM, every hidden layer receives an input sequence that consists of the output sequences of forward and backward layers at the level below.

For Layer 1, the input data are the sequence output of the previous CNN model. Additionally, the output of the last LSTM layer at the terminal time step is adopted as the output of our deep bi-directional LSTM. The advantages of stacking of LSTM layers are two-fold. One is that stacking layers enable the model to learn the characteristic of the raw signal at different time scales. The other is that parameters can be distributed over the space, i.e., layers, instead of increasing memory size, which can contribute to more effective non-linear operations of the input raw signal. The architectures of bi-directional LSTMs and deep bi-directional LSTMs are shown in Figure 4b,c.

### 3.3. Fully-Connected and Linear Regression Layers

In this part, the output representation of the temporal encoder, a two-layer bi-directional LSTMs, is fed into multiple hidden layers to seek a higher-level representation. Two fully-connected dense layers are stacked together, in which the output of one layer is used as the input into the next layer. The computation in each layer is given by:

(11) $$o^{i}=g\left(W_{i}h^{i}+b_{i}\right)$$

where $o^{i}$ and $h^{i}$ denote the output and input of the *i*-th fully-connected layer, respectively. $W_{i}$ and $b_{i}$ represent the transformation matrix and the bias term in the *i*-th fully-connected layer, respectively. The function g() is also set to be ReLu. Additionally, the output of the last layer, $o^{c-1}$, is regarded as the final representation of the input sequence, assuming *c* fully-connected dense layers are successively stacked.

The final learned representation of the raw signal is fed into the final linear regression layer, which predicts the actual tool wear.

### 3.4. Training and Regularization for CBLSTMs

Given the predicted outputs and true targets, the mean squared errors over training data can be calculated and back-propagated to update model parameters. The optimization method named Root Mean Square Propagation(RMSprop)that utilizes the magnitude of recent gradients to normalize the gradients is adopted to optimize model parameters over the objective function [47].

Due to the model complexity of deep learning methods, the large scale of training data is vital for the model’s robust performance. In machine monitoring problems, it is hard to obtain a large scale of training data. Therefore, the regularization technique is applied for our proposed models. Dropout was introduced during model training [48]. Via dropout, parts of the hidden outputs are randomly masked so that these neurons will not influence the forward propagation during training procedures. When it comes to testing phases, the dropout will be turned off, and the outputs of all hidden neurons will have effects on model testing. From another view, dropout can be regarded as an approach to enlarge the training data size. During each training epoch, the application of random masking noise creates novel variants of data samples. In our models, we adopted one dropout layer between LSTM models and the first fully-connected layer and another dropout layer between the first fully-connected layer and the second fully-connected layer. Their masking probabilities are both set to 0.5.

## 4. Experiments

In this section, we empirically evaluated the performances of our proposed CBLSTM. The tool wear monitoring task is conducted. Firstly, the dataset descriptions are given. Then, details about the experimental setup are provided. Finally, the comparison results are shown and discussed.

### 4.1. Descriptions of Datasets

**Figure 5:**
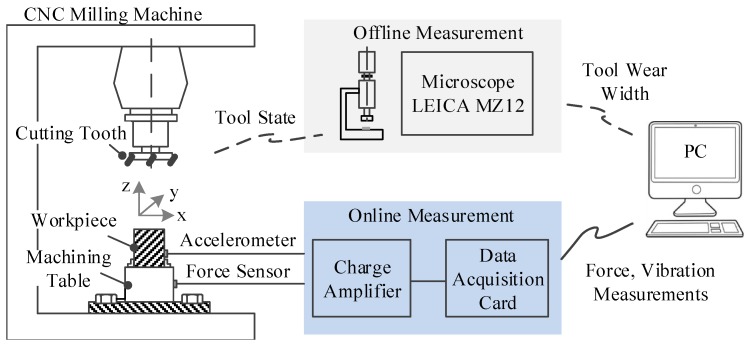
Illustrations for the experimental platform [50].

To experimentally verify the performance of CBLSTM, a high speed CNC machine was run under dry milling operations [49]. The schematic diagram of the experimental platform is shown in Figure 5. The operation parameters are as follows: the running speed of the spindle was 10,400 rpm; the feed rate in the *x* direction was 1555 mm/min; the depth of cut (radial) in the *y* direction was 0.125 mm; the depth of cut (axial) in the *z* direction was 0.2 mm. To acquire data related to this CNC machine’s operation condition, a Kistler quartz 3-component platform dynamometer was mounted between the workpiece and the machining table to measure cutting forces, while three Kistler piezo accelerometers were mounted on the workpiece to measure the machine tool vibration in the *x*, *y* and *z* directions, respectively [49]. Therefore, six different signals acquired by these corresponding six sensors were used in our experiments. DAQ NI PCI1200 was adopted to perform in-process measurements, including force and vibration in three directions (*x*, *y*, *z*) with a continuous sampling frequency of 50 kHz during the tool wear test. Considering that the sampling frequency is quite high such that each data sample has over 100 thousands time steps, the whole sequence is divided into 100 sections, and the max and mean values of each section are kept to form a new time step. By doing this, each data sample is converted into a sequential datum whose length is 100, and the dimensionality of each time step is 12. The corresponding flank wear of each individual flute was measured offline using a LEICA MZ12 microscope after finishing each surface, which is considered to be one cut number in the following data analysis, which will be the target value. Finally, three tool life tests named C1, C4 and C6 were selected as our dataset. Each test contains 315 data samples, while each data sample has a corresponding flank wear. For training/testing splitting, a three-fold setting is adopted such that two tests are used as the training domain and the other one is used as the testing domain. For example, when C4 and C6 are used as the training datasets, C1 will be adopted as the testing dataset. This splitting is denoted as *c1*. The details about training/testing splitting are shown in Table 1. Our task is defined as the prediction of tool wear depth based on the sensory input. To facilitate the training, the target value of training data is firstly scaled into a range [0, 1]. Additionally, the predicted value of testing data will be inverse transformed and then compared to ground-truth values.

### 4.2. Experimental Setup

The following methods will be compared:

* LR: Linear Regression on extracted features of raw signal;
* SVR: Support Vector Regression on extracted features of the raw signal;
* MLP: Multi-layer neural network on extracted features of the raw signal;
* RNN: Basic RNN on the raw signal;
* Deep RNN: A two-layer RNN on the raw signal;
* LSTM: A one-layer LSTM with dropout on the raw signal;
* Deep LSTM: A two-layer LSTM with dropout on the raw signal;
* BLSTM: A bi-directional LSTM with dropout on the raw signal;
* Deep BLSTM: A two-layer bi-directional LSTM with dropout on the raw signal.

Since LR, SVR and MLP cannot address sequential data, feature extraction is conducted firstly. The same setting in [50] was adopted here, and 9 measures (e.g., RMS, variance, wavelet energy, etc.) that are illustrated in Table 2 were extracted from the six sensors. Then, each machine condition can be represented by a 54-dimensional vector, which is fed into the subsequent regression models, including LR, SVR and MLP. LR has no hyperparameter. In SVR, we search the best regularization parameter C from {0.001, 0.01, 0.1, 1, 10}. For MLP, three fully-connected layers with layer sizes of [162, 162, 108] are designed with the activation function as the sigmoid.

Six recurrent models, including RNN, deep RNN, LSTMs, deep LSTMs, bi-directional LSTMs and deep bi-directional LSTMs, are compared. These models can directly process time series data, so that feature extraction is not required here. The input sequence has 100 time steps, and each time step is described by a vector with a dimensionality of 12. For RNN and deep RNN, one-layer with a size of 28 and two layers with sizes of [28,56] recurrent models are fed into two fully-connected layers with a size of [80,100] and the final linear regression layer, respectively. For LSTMs and deep LSTMs, the LSTM layer is replaced with the RNN layer, while the other setting is kept the same as RNN and deep RNN models. Compared to LSTM and deep LSTM, bi-directional processing of input sequences is adopted, and the other settings are kept unchanged.

For our proposed CBLSTM, one-layer CNN is firstly designed, whose filter number, filter size and pooling size are set to 150, 10 and 5. Therefore, the shape of the raw sensory sequence is changed from 100×12 to 19×150 after CNN. Then, a two-layer bi-directional LSTMis built on top of the CNN. Backward and forward LSTMs share the same layer sizes as [150, 200]. Therefore, the output of the LSTM module is the concatenated vector of the representations learned by backward and forward LSTMs, and its dimensionality is 400. Then, before feeding the representation into the linear regression layer, two fully-connected layers with a size of [500, 600] are adopted. The nonlinearity activation functions in our proposed CBLSTM are all set to ReLu.

It should be stated that in our experiment, the selection of the hyperparameter is cross-validated in a portion of training data. To quantitatively evaluate the performances of all compared models, two measures are adopted, including Mean Absolute Error (MAE) and Root Mean Squared Error (RMSE). MAE is the average value of the absolute values of the errors. RMSE is the square root of the average of the square of all of the errors. The corresponding equations for the calculations of these two measures are given as follows:

(12) $$MAE=\frac{1}{n}\sum_{i=1}^{n}\left|{\bar{y}}_{i}-y_{i}\right|$$

(13) $$RMSE=\sqrt{\frac{1}{n}\sum_{i=1}^{n}{\left({\bar{y}}_{i}-y_{i}\right)}^{2}}$$

where $y_{i}$ and ${\bar{y}}_{i}$ are true and predicted tool wear depth.

### 4.3. Experimental Results on Tool Wear Prediction

In this section, we show a comparison of LSTMs with several benchmark methods. Additionally, MAE and RMSE of all methods on three different datasets are shown in Table 3 and Table 4, respectively.

We firstly observed that among regression models, including LR, SVR and MLP based on expert features, LR performs worst due to the limitation of linear models. SVR with the RBF kernel and MLP with the sigmoid activation function are both nonlinear models that can capture the nonlinear relationships between the expert features and the tool wear. However, recurrent models based on the raw input signal all outperform these models based on expert features. It has been shown that recurrent neural network models are able to learn meaningful representations from the raw signal without any feature engineering.

Among recurrent models, deep models that contain two hidden recurrent layers always perform better than their corresponding normal models that only contain one hidden recurrent layer. It is shown that deep models are able to learn more abstract and discriminative representation due to stacked hidden layers. When it comes to different recurrent units, including basic recurrent, LSTM and bi-directional LSTM ones, bi-directional LSTM performs the best, and the basic recurrent model performs the worst. It is obvious that basic LSTMs perform slightly better than RNN. The reasons may be the fact that gate functions employed in LSTMs can enable it to capture long-term dependency better than RNN. Additionally, the bi-directional structure can discover future information compared to the forward recurrent structure.

It is shown that our proposed CBLSTM model achieves the best performance among all compared methods. Compared to the most competitive model, deep BLSTMs, CBLSTM adopts convolutional neural network to address the raw signal and then builds recurrent modules on top of CNN. The experimental results have verified the effectiveness of the convolutional operation in our proposed CBLSTMs. The CNN is adopted to extract local features, which can filter the noise in the raw signal effectively. In addition, CNN can also reduce the length of sequential data. In our case, the length of raw sequential data is reduced from 100 to 19, and the short sequential data can be more easily captured by the following recurrent model.

**Figure 6:**
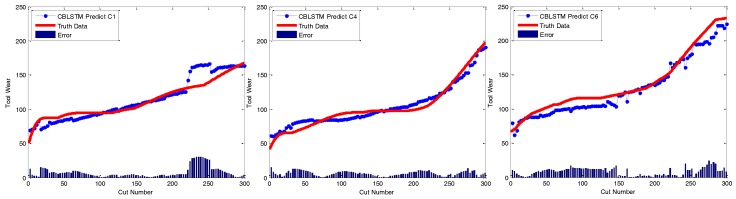
Regression analysis of CBLSTM for three different testing scenarios including C1, C4 and C6.

At last, to qualitatively demonstrate the effectiveness of CBLSTM models, the predicted tool wears under different datasets are illustrated in Figure 6. The actual tool wear conditions measured offline by a microscope are also displayed, respectively. It is found that the predicted tool wear overall is able to follow the trend of the groundtruth data well.

Our proposed CBLSTM was conducted using a NVIDIA Tesla K40c GPU on a Windows Server with a dual-2.70-GHz CPU and a RAM of 512 GB. The training time for one epoch is 5 s, and the testing time for each sample of our algorithm is only 0.027 s, so that CBLSTM can be an effective solution for real-time machine monitoring.

### 4.4. Effects of Dropout and Bi-Directional Modules on the Performances of CBLSTM

In the CBLSTM model, two key modules are adopted, including dropout and bi-directional recurrent structures. In the following, their effectiveness is experimentally investigated.

**Figure 7:**
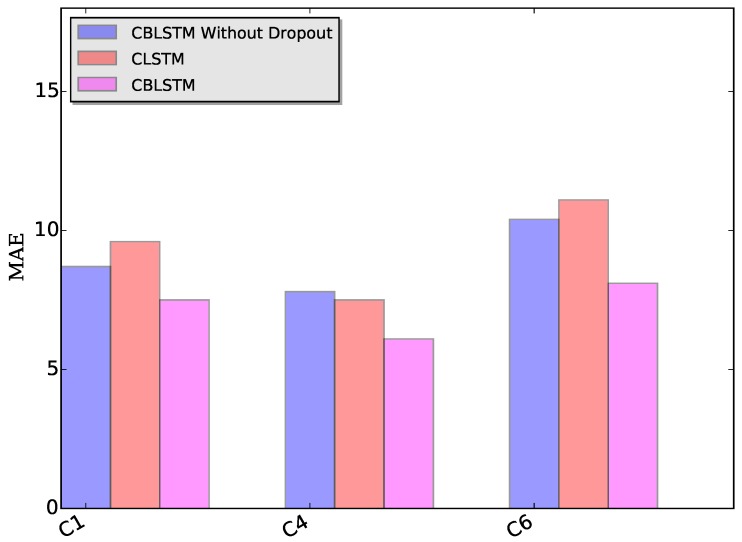
MAE measures of our proposed CBLSTMs without dropout, CLSTM and CBLSTM under three different datasets: C1, C4 and C6, respectively.

**Figure 8:**
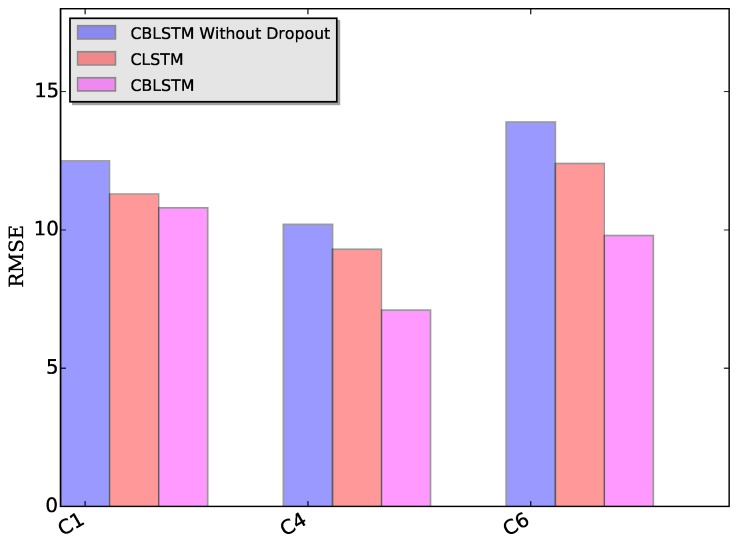
RMSE measures of our proposed CBLSTMs without dropout, CLSTM and CBLSTM under three different datasets: C1, C4 and C6, respectively.

**Figure 9:**
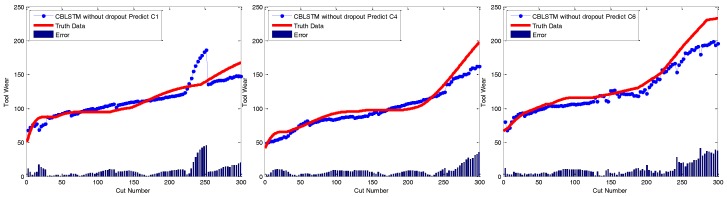
Regression analysis of CBLSTM without dropout for three different testing scenarios including C1, C4 and C6.

Dropout module: The applied dropout layer can relieve the possible overfitting problem. To verify the effectiveness of the dropout module, CBLSTM without dropout was run on these three datasets. The MAE and RMSE measures are shown in Figure 7 and Figure 8. Additionally, the predicted tool wears under different datasets are illustrated in Figure 9. It is shown that the dropout operations are able to reduce the regression error of our CBLSTM method.

**Figure 10:**
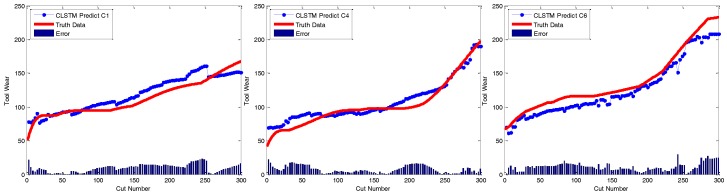
Regression analysis of CLSTM for three different testing scenarios including C1, C4 and C6.

Bi-directional module: The adopted bi-directional module can enable our CBLSTM method to consider the previous and future context of each time step. Here, the performances of Convolutional LSTM networks (CLSTM) were evaluated, and the corresponding MAE and RMSE measures are shown in Figure 7 and Figure 8. Compared to CBLSTM, CLSTM uses normal LSTM as the temporal encoder, and other hyperparameters are set to be the same as CBLSTM. Further, the tool wears predicated by CLSTM under different datasets are illustrated in Figure 10. Experimental results state that the bi-directional structure is able to improve the regression performance.

## 5. Conclusions

In this work, CBLSTM has been proposed to address tool wear prediction tasks. In CBLSTM, CNN is firstly designed to extract local features from raw sequential data. Then, a bi-directional LSTM is adopted to encode the temporal information. As an advanced recurrent model, bi-directional LSTMs are able to capture long-term dependencies in forward and backward ways. Additionally, the stacked LSTM layers can enable our module to learn more abstract and deep features. It is shown that CBLSTM does not require any expert knowledge and feature engineering. In the task of tool wear prediction, experimental results have verified the superior performance of our CBLSTM method. Therefore, our proposed CBLSTM is able to capture and discover meaningful features under the sensory signal for machine health monitoring.

In future work, we plan to introduce wavelet transformation, which is an effective tool to analyze the machine sensory signal, into the deep neural network models. The combination of shallow feature extraction and deep feature extraction methods may be more effective and efficient in the area of machine health monitoring.

## Figures and Tables

**Table 1:** Configurations for training/testing splitting.

Symbol	Training Domain	Testing Domain
*c1*	C4, C6	C1
*c4*	C1, C6	C4
*c6*	C1, C4	C6

**Table 2:** List of extracted features.

Domain	Features	Expression
Statistical	RMS	$z_{rms}=\sqrt{\frac{1}{n}\sum_{i=1}^{n}z_{i}^{2}}$
	Variance	$z_{var}=\frac{1}{n}\sum_{i=1}^{n}{\left(z_{i}-\bar{z}\right)}^{2}$
	Maximum	$z_{max}=max\left(z\right)$
	Skewness	$z_{skew}=E\left[{\left(\frac{z-\mu }{\sigma }\right)}^{3}\right]$
	Kurtosis	$z_{kurt}=E\left[{\left(\frac{z-\mu }{\sigma }\right)}^{4}\right]$
	Peak-to-Peak	$z_{p-p}=max\left(z\right)-min\left(z\right)$
Frequency	Spectral Skewness	$f_{skew}=\sum_{i=1}k{\left(\frac{f_{i}-\bar{f}}{\sigma }\right)}^{3}S\left(f_{i}\right)$
	Spectral Kurtosis	$f_{kurt}=\sum_{i=1}k{\left(\frac{f_{i}-\bar{f}}{\sigma }\right)}^{4}S\left(f_{i}\right)$
Time-Frequency	Wavelet Energy	$E_{WT}=\sum_{i=1}^{N}wt_{\phi }^{2}\left(i\right)/N$

**Table 3:** MAE for compared methods on these three datasets. Bold face indicates the best performances. RNN, Recurrent Neural Network.

Category	Methods	Datasets		
		*c1*	*c4*	*c6*
Regression Models	LR	24.4	16.3	24.4
	SVR	15.6	17.0	24.9
	MLP	24.5	18.0	24.8
Recurrent Models	RNN	13.1	16.7	25.5
	Deep RNN	7.8	9.4	19.3
	LSTMs	19.6	15.6	25.3
	Deep LSTMs	8.3	8.7	15.2
	BLSTMs	9.9	10.8	15.7
	Deep BLSTMs	8.7	6.7	14.4
Our Model	CBLSTMs	7.5	6.1	8.1

**Table 4:** RMSE for compared methods on these three datasets. Bold face indicates the best performance.

Category	Methods	Datasets		
		*c1*	*c4*	*c6*
Regression Models	LR	31.1	19.3	30.9
	SVR	18.5	19.6	31.5
	MLP	31.2	20.0	31.4
Recurrent Models	RNN	15.6	19.7	32.9
	Deep RNN	12.5	11.8	22.9
	LSTMs	23.9	20.8	32.4
	Deep LSTMs	12.1	10.2	18.9
	BLSTMs	12.3	14.7	20.8
	Deep BLSTMs	11.5	9.1	18.9
Our Model	CBLSTMs	10.8	7.1	9.8
